# Social Media: An Innovative and Effective Tool for Educational and Research Purposes of the Pharmaceutical and Medical Professionals

**Published:** 2018

**Authors:** Fanak Fahimi


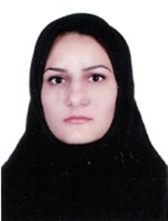


“Seek knowledge from the cradle to the grave.” is one of the first established sayings that means acquiring knowledge is an open end path. The saying “Learn the knowledge, though in China”, means “the pursuit of knowledge is obligatory for any Muslim anywhere”.

Todays, students live in social media where they find the instinctual needs to connect with each other. Socializing has been always around in one form or another and the majority of times in the groups of similar harmonious people which different subjects of interests are shared. Media includes the tools and technologies to make connections such as TV, radio, websites, photos, and drawings. More advanced type of media are internet based websites and applications. An educational social network is a group of people who use media for the purpose of learning. The amount of interaction and participation could differ to a great extent among followers and the members of a network.

Many of the academic social networking have been approved and established for academic purposes. Members can make profiles, create or join scientific and medical discussion groups and channels, share pictures, audios, voice memos, and videos. Potential of having a profound impact on virtual practices for teaching objectives exists. Social media enables the faculties to facilitate learning for the students to conduct surveys within the members of a group with common goals and/or backgrounds. We are proposing that social media can be benefiting pharmacists or other health care providers in their educational journey.

- There are several advantages using social media as an educational gizmo. It is without boundaries, not expensive, efficient, provides more access to specialized experts, unlimited resources, friendly relations, prompt and reliable surveys, abreast, and decent tool for creating assignments.

- It is not expensive. Most of the websites or applications of social media are either free or very inexpensive.

- It is more efficient. Teachers can apply different presentation techniques such as audios, video clips, interactive Q & As. Students will be equipped to lead and solve problems for any comparable paradigm.

- It provides more access to specialized experts in a specific field. General knowledge about a clinical subject could be replaced by more specialized knowledge through professional social networks.

- It enables users to access unlimited resources. Social platforms are the best retrievable and searchable archives and libraries. Almost every post are saved there for a long period of time, unlike papers, PowerPoint presentations, and audios.

- Surfing in the academic social media creates more friendly relationships. Online programs enable instructors to feel closer to the learners who are mainly from younger generation and have boundary barriers. Many students are reluctant to ask questions in the classroom environment. Yet, they become more interactive in the social media.

- It facilitates prompt and reliable surveys. Moreover, surveys, reviews, audits, and assessments are easily performed by vote robots in such platforms. While the results of those investigations are more reliable and acceptable due to the fact that it is shown in front of the participants’ eyes.

- It keep users abreast. Considering the constant evolving medical world, one cannot keep pace with this information explosion. An average book takes anywhere from 1 to 3 years to be published. By then, it may require revision due to rapid updates in the medical fields. Whereas, learners can access new updates by simply search in the related sites and apps.

- It does not require traveling thousands miles away for education. Traditionally, a discussion panel only takes place physically in the morning reports, journal clubs, and designated places or times. Though, a group chat with colleagues in similar profession via social media is practical with no such limitations.

- It is a decent tool for assignments. Tasks could be posted online by the faculty members. Some apps enable professors to save a draft of the created task and schedule it to be published at a later time. The students can submit their completed work using the same method.

- There is a few disadvantages using social media for educational drive as well. It requires knowledge of information technology (IT), verification by the subscribers, ability to locate similar professionals. Other likely barriers are internet crash, and time consuming trust forming.

- Insufficient information technology (IT) knowledge for older generation faculties could be a challenge and make them uneasy using cyberspace for the educational purposes.

- Verification by the subscribers is essential when using a public source. The chief drawback of social media is that a scientific subject can be broadcasted anonymously or by an individual without proper scientific knowledge. Mainly, there is no systematic moderation and control over the distribution of the incorrect posts. Unprofessional administrators may misguide the users. Supposedly, most social media should follow specific regulations; for example they are forced to remove incorrect information when reported by the users. Could these regulations guarantee accurate data throughout a network? Needless to say that even a classroom setting is never flawless and immaculate. Even though, this seems to be a big issue, usually physicians and pharmacists have the vision to distinguish and use more creditable and reliable sources.

- Reaching out the professionals in the same field is sometimes requires long period of time for both instructors and students. The question is how these newer approaches can be utilized efficiently to create a connection amongst the members to connect to the tutorial presented.

- Internet crash or absence of internet access in some areas should not be underestimated.

- Building trust may be slow and time consuming. Interactive debate in a group with a specific goal starts only if the members are certain about the credibility of the professionals. Consequently, it will last longer and lead to much more learning chances.

Social methods are considered a favorable tool even for younger students. Social media sites are applied by school teachers around the world such as Twiducate, Twitter, Skype, Blackboard, Wiki Classrooms, Edmodo and MinecraftEdu. Many have added features as video games to engage 

students further.

Several social media play major roles in professional communications. LinkedIn, Facebook, Twitter, Instagram and Telegram messenger are the most powerful and popular social podium amongst pharmacists and physicians in many countries. 

For example, LinkedIn is a professional application and database with over 25 million members who are mostly male and university graduates. Instagram is a content sharing platform containing inspirational pictures and short videos that can give informational messages, useful for a quick view. Telegram messenger has become a dominant and inevitable element of Iranian university students’ lives in the recent years. However, the number of Iranian pharmacists, or medical affiliates who use this media for learning and education has not been documented yet. 

To summarize, in the fast growing medical era, it is not realistic nor just to deprive students and professionals from this mind-blowing technology. The positive aspects weigh more than the negative ones.

*Fanak Fahimi is Professor of Clinical Pharmacy in Department of Clinical Pharmacy, School of Pharmacy, Shahid Beheshti University of Medical Sciences, Tehran, Iran. She could be reached at the following e-mail addresses: fanakfahimi@yahoo.com; fahimi@sbmu.ac.ir*

